# Biomarkers used in the diagnosis and prognosis of gastric cancer in young patients: a scientometric analysis

**DOI:** 10.3389/fmed.2025.1586742

**Published:** 2025-04-09

**Authors:** Nurbek Kozhakhmetuly Azbergenov, Saule Zhumabaevna Akhmetova, Talshyn Amirkhanovna Nurulla, Abdiraman Rsalievich Kaliev, Aigul Bulatovna Ramankulova, Anar Balkashevna Tulyayeva, Nurgul Meirimovna Kereeva

**Affiliations:** ^1^Department of Pathological Anatomy and Forensic Medicine, West Kazakhstan Marat Ospanov Medical University, Aktobe, Kazakhstan; ^2^Department of Oncology, Medical Center of West Kazakhstan Marat Ospanov Medical University, Aktobe, Kazakhstan

**Keywords:** stomach cancer, biomarkers, adults, young, immunohistochemistry

## Abstract

**Introduction:**

Gastric cancer in young people is a global health burden, although it is less common than in other age groups. The use of biomarkers is developing in the diagnosis, treatment selection and prognosis of gastric cancer in young patients. In this bibliometric analysis we aim to evaluate the progress of this knowledge, trend topic development and scientific teams and countries involvements in the topic of biomarkers role in gastric cancer in young patients.

**Methods:**

The data were obtained from Scopus (536 publications) for the period 1993–2024, all relevant metadata were analyzed using RStudio and Biblioshiny package to perform global trends and hotspots analysis.

**Results:**

Publication trends show a constant increase in interest in gastric cancer biomarkers used in the diagnosis and prognosis of gastric cancer in young patients (7.71% per year). The leading countries were China, USA, and Japan, between which there is strong and sustained collaborations. International co-authorship is relatively low (19.4%). The most prolific research centers were Sungkyunkwan University, Sun Yat-sen University, and Fudan University. The most productive researchers were Zhang X., Wang Y., and Li Y. Keywords analysis showed an increase in mentions of topics related to diagnostics (biomarkers, immunohistochemistry), personalized medicine and prognosis.

**Conclusion:**

Bibliometric analysis of more than three decades research articles on gastric cancer biomarkers in young patients showed a steady increase, with strong contributions from leading countries and institutions, highlighting the growing focus on diagnostics, personalized medicine, and prognosis.

## 1 Introduction

Gastric cancer is one of the major global health burdens, ranking fifth in incidence and fourth in mortality worldwide (1, 2). Gastric cancer in young people (aged ≤45 years) accounts for less than 10% of all new cases (3). Gastric cancer has different age characteristics depending on the region. In most countries, it is considered a disease of old age, and its prevalence increases with age. Although the overall incidence of gastric cancer has decreased in recent decades and is projected to continue to decrease, many epidemiological studies in the East, West (4, 5) and USA (6) showed an increasing trend in morbidity. Due to the lack of obvious symptoms and rapid tumor progression, diagnosis and treatment are often delayed in young cancer patients (7). GC in young patients has more aggressive biological behavior than in middle-aged and elderly patients (8). A significant proportion of young patients often have a more advanced stage of the disease compared to older patients (3), and the prognosis remains unfavorable (9).

It is known that the clinical and pathological features of young patients with gastric cancer differ from those of older patients. Patients with gastric cancer aged 40 years and younger exhibit a number of distinctive characteristics compared to patients in the older age group (over 40 years). In particular, they are more often women, have more aggressive forms of the disease, are more likely to have undifferentiated and diffuse tumor types, and have a positive family history of cancer. In addition, this group has an increased rate of recurrence in the remaining part of the stomach (10). Younger patients diagnosed with gastric cancer are more likely to have tumors with signet ring cell or diffuse histology, metastatic disease, and germline mutations in the CDH1 gene compared with older patients (11). Wang et al. talk about the exclusive location of SDH-deficient GIST tumors in the stomach, the manifestation of a predisposition to children and young people with a predominance of the female sex (12). However, despite this, 5-year survival in young patients is higher than in older patients (13). With the advent of the era of personalized cancer treatment, the lack of biological information such as morphology, size, and enhancement of lesions, which are traditionally assessed by visual examination and do not take into account tumor heterogeneity, is becoming apparent. This deficit is gradually being filled by biomarkers that provide more accurate molecular and genetic profiling of tumors, improving diagnosis and the selection of personalized therapies (14, 15).

A large number of prognostic biomarkers in gastric cancer have been identified thanks to the development of bioinformatics methods (16, 17). As already mentioned, gastric cancer in its early stages has no specific clinical signs, and most patients consult a doctor at a middle or late stage, which leads to an unfavorable prognosis. Therefore, it is extremely important to study the mechanisms of gastric cancer and find biomarkers for its early detection (18) and forecast. There is also an urgent need to develop new biomarkers that will allow physicians to more accurately predict the course of the disease and make informed decisions regarding therapy. The use of several biomarkers together facilitates a more comprehensive assessment of the patient’s condition and can significantly improve the accuracy of predicting disease outcomes (2).

Bibliometric analysis helps to achieve a qualitative and quantitative analysis of publications devoted to the study of biomarkers used in the diagnosis and prognosis of gastric cancer in young patients, using a multidisciplinary approach to sort out research areas and understand the trends and directions of research (19).

Overall, bibliometric analysis provides valuable information about the publication landscape devoted to this topic. The aim of this bibliometric analysis is to review the global scientific literature on biomarkers used in the diagnosis and prognosis of gastric cancer in young patients over the past 31 years. The study aims to systematize existing knowledge, identify current global publication trends, leading authors and most cited references, as well as to analyze the current state and summarize research hot spots. This study effectively represents a comprehensive global roadmap for navigating future research in this area.

## 2 Methods

### 2.1 Eligibility criteria and data source

The analysis included only original research articles published in English. The data were obtained from the Scopus database, and all relevant metadata were extracted in BibTeX format. The data presented in the accompanying BibTeX file (Table 1) were subsequently analyzed using RStudio 2024.12.0.

**Table 1:** Queries used to search the Scopus database.

Code	Queries
#1	“Biomarker” OR “Marker, Biological” OR “Biological Marker” OR “Markers, Biological” OR “Biological Markers” OR “Biologic Markers” OR “Markers, Biologic” OR “Biologic Marker” OR “Marker, Biologic” OR “Markers, Clinical” OR “Clinical Marker” OR “Marker, Clinical” OR “Clinical Markers” OR “Surrogate Markers” OR “Marker, Surrogate” OR “Surrogate Marker” OR “Markers, Surrogate” OR “Surrogate Endpoints” OR “Endpoints, Surrogate” OR “Surrogate End Points” OR “End Points, Surrogate” OR “Surrogate Endpoint” OR “Endpoint, Surrogate” OR “Surrogate End Point” OR “End Point, Surrogate” OR “Markers, Immunologic” OR “Immune Marker” OR “Marker, Immune” OR “Immune Markers” OR “Markers, Immune” OR “Immunologic Marker” OR “Marker, Immunologic” OR “Immunologic Markers” OR “Markers, Laboratory” OR “Laboratory Marker” OR “Marker, Laboratory” OR “Laboratory Markers” OR “Serum Markers” OR “Marker, Serum” OR “Serum Marker” OR “Markers, Serum” OR “Viral Markers” OR “Viral Marker” OR “Marker, Viral” OR “Markers, Viral” OR “Biochemical Marker” OR “Marker, Biochemical” OR “Biochemical Markers” OR “Markers, Biochemical”
#2	“Neoplasm, Stomach” OR “Stomach Neoplasm” OR “Gastric Neoplasms” OR “Gastric Neoplasm” OR “Neoplasm, Gastric” OR “Neoplasms, Gastric” OR “Neoplasms, Stomach” OR “Cancer of Stomach” OR “Stomach Cancers” OR “Cancer of the Stomach” OR “Gastric Cancer” OR “Cancer, Gastric” OR “Cancers, Gastric” OR “Gastric Cancers” OR “Stomach Cancer” OR “Cancers, Stomach” OR “Cancer, Stomach” OR “Gastric Cancer, Familial Diffuse”
#3	“Adults, Young” OR “Adult, Young” OR “Young Adults”
#4	#1 and #2 and #3

### 2.2 Search strategy

#### 2.2.1 Source of data used in the analysis

**Figure 1:**
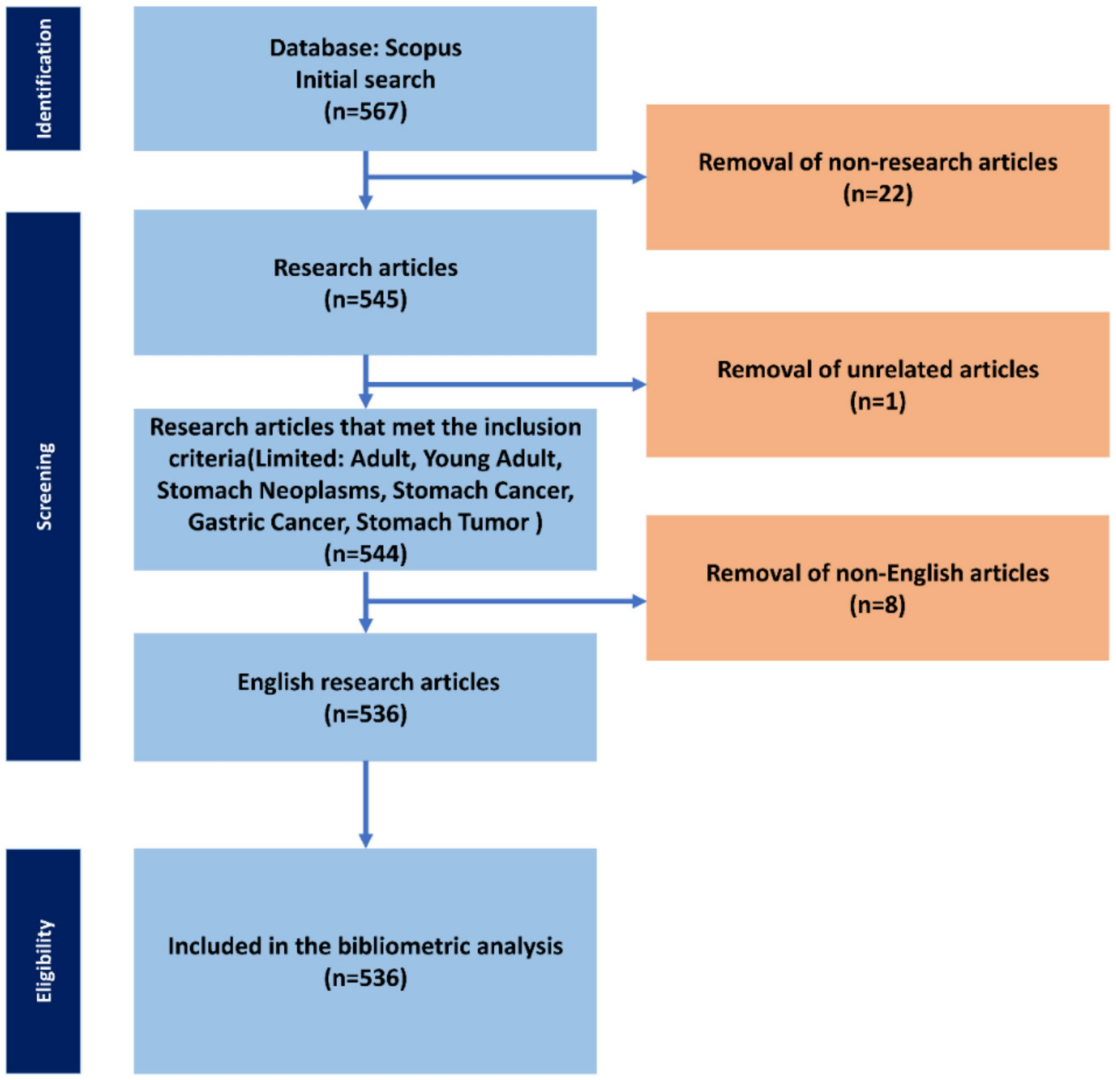
Flow chart of selection of research articles of biomarkers used in diagnosis and prognosis of gastric cancer in young patients.

In this research study, an extensive search was conducted in Scopus, which was chosen as the main and only database. We used combinations of Boolean and wildcard search operators to identify relevant keywords for our study (Table 1). The search was conducted in November 2024, and the comprehensive search strategy is outlined in Figure 1. Articles that were considered irrelevant based on their failure to meet the inclusion and exclusion criteria were systematically excluded.

#### 2.2.2 Inclusion and exclusion criteria

We applied the following inclusion criteria: research articles, articles containing the keywords: Adult, Young Adult, Stomach Neoplasms, Stomach Cancer, Gastric Cancer, Stomach Tumor, articles in English. Exclusion criteria: articles that did not meet the inclusion criteria, articles not in English. After this process, a total of 536 research articles were imported for bibliometric analysis.

### 2.3 Bibliometric analysis

Data management and bibliometric analysis were performed using the Biblioshiny package (RStudio 2024.12.0 + 467 “Kousa Dogwood”). The analysis covered a 31-year period, focusing exclusively on research articles. Top institutions were identified based on the volume of publications related to biomarkers used in the diagnosis and prognosis of gastric cancer in young patients.

In addition, we constructed collaborative networks involving 1,226 research institutions for research articles. These networks were developed using clustering and data normalization algorithms applied to 536 research articles, guided by the association parameter. Authors with significant contributions were identified based on the frequency of their published articles. The analysis also included identifying the 10 most frequently cited articles and the top journals in the field. This study further explored the interactions between 10 key journals, research areas and countries that contributed to the study of biomarkers used in the diagnosis and prognosis of gastric cancer in young patients over the past 31 years. To visualize the global scientific collaborations, the connections were presented on a world map. The tree map visualization highlighted the 10 most frequently used keywords in publications on the topic. Finally, the thematic structure of the research was also examined.

## 3 Results

### 3.1 Key characteristics of the review of retrieved research articles

We initially retrieved 567 articles from the Scopus database. After applying the eligibility criteria and excluding 31 ineligible studies, we considered a total of 536 research articles for our bibliometric analysis (Figure 1). Scientific articles from 211 sources were analyzed. The annual growth rate of 7.71% is moderately high, which reflects the sustainable interest of the scientific community in new discoveries and funding of research of biomarkers used in the diagnosis and prognosis of gastric cancer in young patients. The rate of international co-authorship is relatively low (19.4%), indicating a relatively low trend of international collaboration in this area, which requires increase. A high degree of collectivity is noticeable (9.64 co-authors per document), which is most likely due to the fact that research in this area is often carried out by large interdisciplinary groups. The study of biomarkers used in the diagnosis and prognosis of gastric cancer in young patients has a sustainable interest and has already achieved a stable level of publication activity, as evidenced by the average document age of 7.85 years. The indicator of 53.56 citations per article per year is quite high, which indicates the preservation of high scientific significance of research of biomarkers used in the diagnosis and prognosis of gastric cancer in young patients. Research articles written by US researchers dominate in terms of average citation rates with 4,279 and 12,345 citations per article per year, respectively. The US has traditionally been a leader in many scientific fields, and the high citation rate of these publications is an indicator of their influence and importance at the global level in this research area. A total of 3,691 authors from 57 countries participated in the 536 research studies included in the analysis. This shows that the study of biomarkers used in the diagnosis and prognosis of gastric cancer in young patients has attracted the attention of scientists from different parts of the world, which may indicate the global importance of the considered field. However, a higher percentage of international co-authorship could highlight an even greater degree of interaction between countries, which is not observed. A large set of 1,188 author keywords and their analysis can provide important insights into the structure of studies, the main topics and directions in the scientific field, as well as possible points for further study and improvement.

### 3.2 Trends in publications and citations

There is marked variability in the number of publications on this topic over the 31-year period from 1993 to 2024, which may indicate different stages of scientific interest, economic factors, or changes in the priorities of research groups. In particular, at the beginning (1993 and 1994) minimal figures were recorded – one article per year, which may indicate the initial stage of studying this topic. The period from 1995 to 2007 is characterized by a sharp decline in the number of publications, which may be due to limited attention to biomarkers in the context of gastric cancer, insufficient development of the field, or priority given to other scientific areas. The smallest number of scientific publications occurred in 1993, 1994, 2008, 2023, during these years from 1 to 7 scientific articles were published per year. Since 2014, there has been a significant increase in the number of publications, as evidenced by two peaks in 2014 (59 publications) and 2020 (57 publications), indicating a resurgence of interest in this topic (Table 2). This may be due to improvements in diagnostic technologies, increased awareness of the importance of biomarkers, and the development of new therapeutic methods.

**Table 2:** Global annual citation rates of research articles of biomarkers used in the diagnosis and prognosis of gastric cancer in young patients.

Year	Mean total citation per article	Number of articles published	Mean total citation per year	Citable years
1993	20	1	0.62	32
1994	46	1	1.48	31
2008	37.5	4	2.21	17
2009	53.64	14	3.35	16
2010	40.06	16	2.67	15
2011	53.4	25	3.81	14
2012	25.22	23	1.94	13
2013	30.98	46	2.58	12
2014	165.42	59	15.04	11
2015	46.95	44	4.7	10
2016	41.32	53	4.59	9
2017	26.43	42	3.3	8
2018	99.34	35	14.19	7
2019	46.06	49	7.68	6
2020	30.58	57	6.12	5
2021	22.33	46	5.58	4
2022	13.43	7	4.48	3
2023	7.25	4	3.62	2
2024	0.7	10	0.7	1

The ranking of the 10 most cited publications (Table 3) shows that the greatest contribution to the study of biomarkers used in the diagnosis of gastric cancer in young people was made by works published in 2014. Article by Herbst et al. (2014) on prognostic correlates of response to the anti-PD-L1 antibody MPDL3280A was the most cited, receiving 4,279 citations. This highlights the key role played by work focused on therapeutic aspects, such as the use of immune agents. An article on circulating tumor DNA, also published in 2014, it ranked second with 3,683 citations, demonstrating the growing interest in molecular diagnostic methods at that time.

**Table 3:** Ranking of the 10 most cited scientific publications devoted to the study of the role of biomarkers in the diagnosis and prognosis of gastric cancer in young patients.

Ranking	Study references	Title of the document	Journal name	Total citations	DOI
1	Herbst et al., 2014	Predictive correlates of response to the anti-PD-L1 antibody MPDL3280A in cancer patients	Nature	4,279	10.1038/nature14011
2	Bettegowda et al., 2014	Detection of circulating tumor DNA in early-and late-stage human malignancies	Science Translational Medicine	3,683	10.1126/scitranslmed.3007094
3	Fuchs et al. 2018	Safety and efficacy of pembrolizumab monotherapy in patients with previously treated advanced gastric and gastroesophageal junction cancer: phase 2 clinical keynote-059 trial	JAMA Oncology	1,423	10.1001/jamaoncol.2018.0013
4	Kim et al., 2018	Comprehensive molecular characterization of clinical responses to PD-1 inhibition in metastatic gastric cancer	Nature Medicine	1,123	10.1038/s41591-018-0101-z
5	Hansford et al., 2015	Hereditary diffuse gastric cancer syndrome: CDH1 mutations and beyond	JAMA Oncology	545	10.1001/jamaoncol.2014.168
6	Hecht et al., 2016	Lapatinib in combination with capecitabine plus oxaliplatin in human epidermal growth factor receptor 2–positive advanced or metastatic gastric, esophageal, or gastroesophageal adenocarcinoma: TRIO-013/LOGiC— a randomized phase III Trial	Journal of Clinical Oncology	482	10.1200/JCO.2015.62.6598
7	Chen et al., 2020	Non-invasive early detection of cancer four years before conventional diagnosis using a blood test	Nature Communications	399	10.1038/s41467-020-17316-z
8	Park et al., 2015	Phase III trial to compare adjuvant chemotherapy with capecitabine and cisplatin versus concurrent chemoradiotherapy in gastric cancer: final report of the adjuvant chemoradiotherapy in stomach tumors trial, including survival and subset analyses	Journal of Clinical Oncology	378	10.1200/JCO.2014.58.3930
9	Wang et al., 2019	Safety, efficacy and tumor mutational burden as a biomarker of overall survival benefit in chemo-refractory gastric cancer treated with toripalimab, a PD-1 antibody in phase Ib/II clinical trial NCT02915432	Annals of Oncology	331	10.1093/annonc/mdz197
10	Pietrantonio et al., 2019	Individual patient data meta-analysis of the value of microsatellite instability as a biomarker in gastric cancer	Journal of Clinical Oncology	308	10.1200/JCO.19.01124

Global annual citation rates of articles studying the use of biomarkers in the gastric cancer field show significant fluctuations: In 2009, 53.64 citations per article were recorded, which also confirms the high interest in the research during that period. However, starting from 2015, the citation rate began to decrease, which may indicate the completion of major studies on this topic and the lack of new publications with high scientific impact (Table 2).

The trend of average annual citations shows significant variability with periodic fluctuations from 1993 to 2017. There is a gradual decline from 2018 to 2024, with citation rates falling sharply from 14.19 citations to 0.70 citations. This may indicate a decline in interest in the field or the exhaustion of key studies that have been heavily cited over a long period of time. Notably, the highest average annual citation rate of 15.04 citations per year was observed in 2014, which dropped sharply in 2015 to 4.7 and then showed a declining trend until 2017 (Table 2).

**Figure 2:**
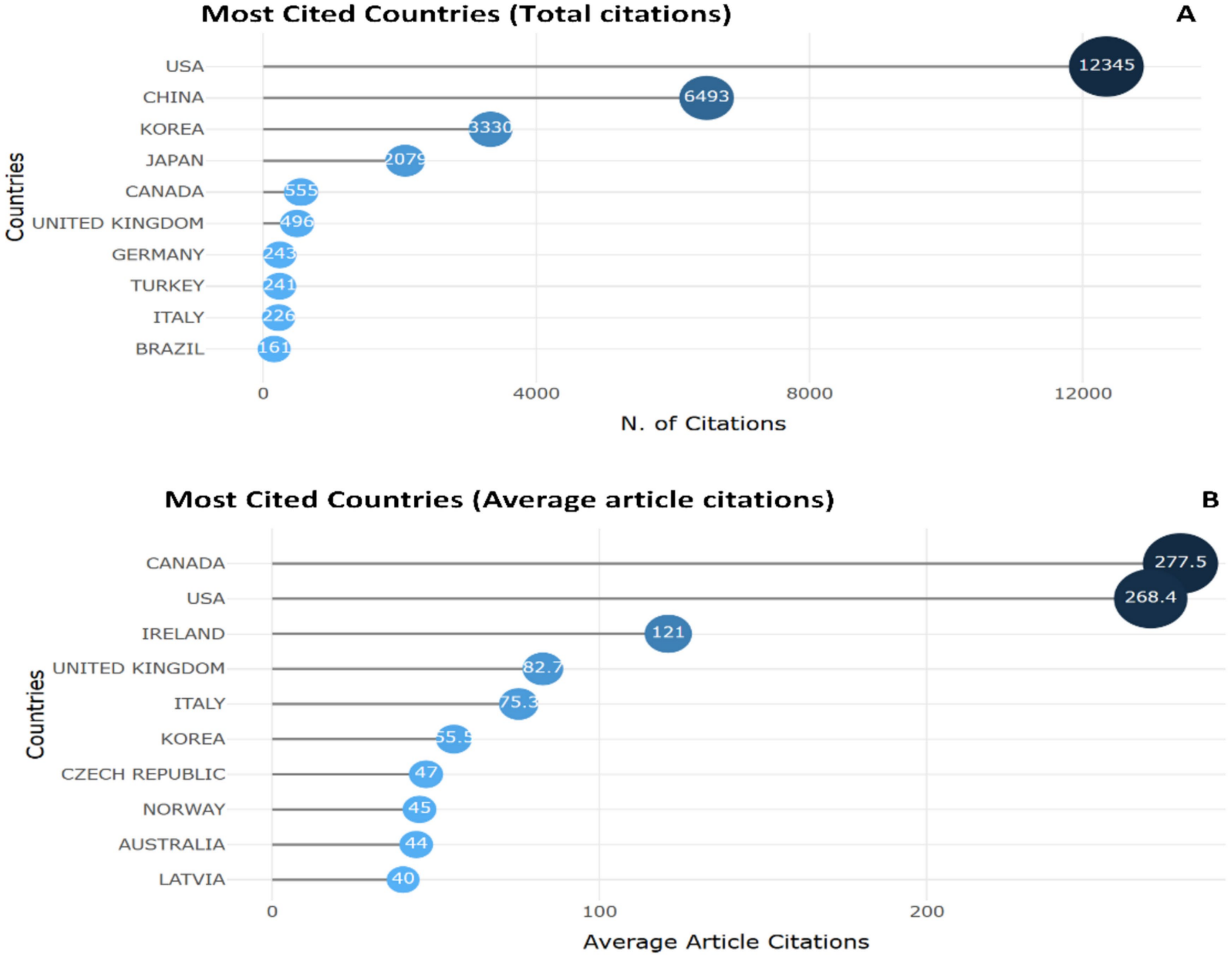
Top 10 most cited countries. **(A)** According to overall citations and **(B)** according to average article citations publishing papers on the use of biomarkers in the diagnosis and prognosis of gastric cancer in young patients.

The leader in Total Citations is the United States (12,345 citations), reflecting the strong impact of research in this country (Figure 2A). It is followed by China with 6,493 citations and Korea with 3,330 citations. The Average Article Citations is highest in Canada (277.50), indicating the high quality and impact of published papers despite their smaller number. Among Asian countries, China and Korea are the most productive, but China has a lower average citation count (32.00) compared to Korea (55.50) (Figure 2B).

### 3.3 Most relevant affiliations

**Figure 3:**
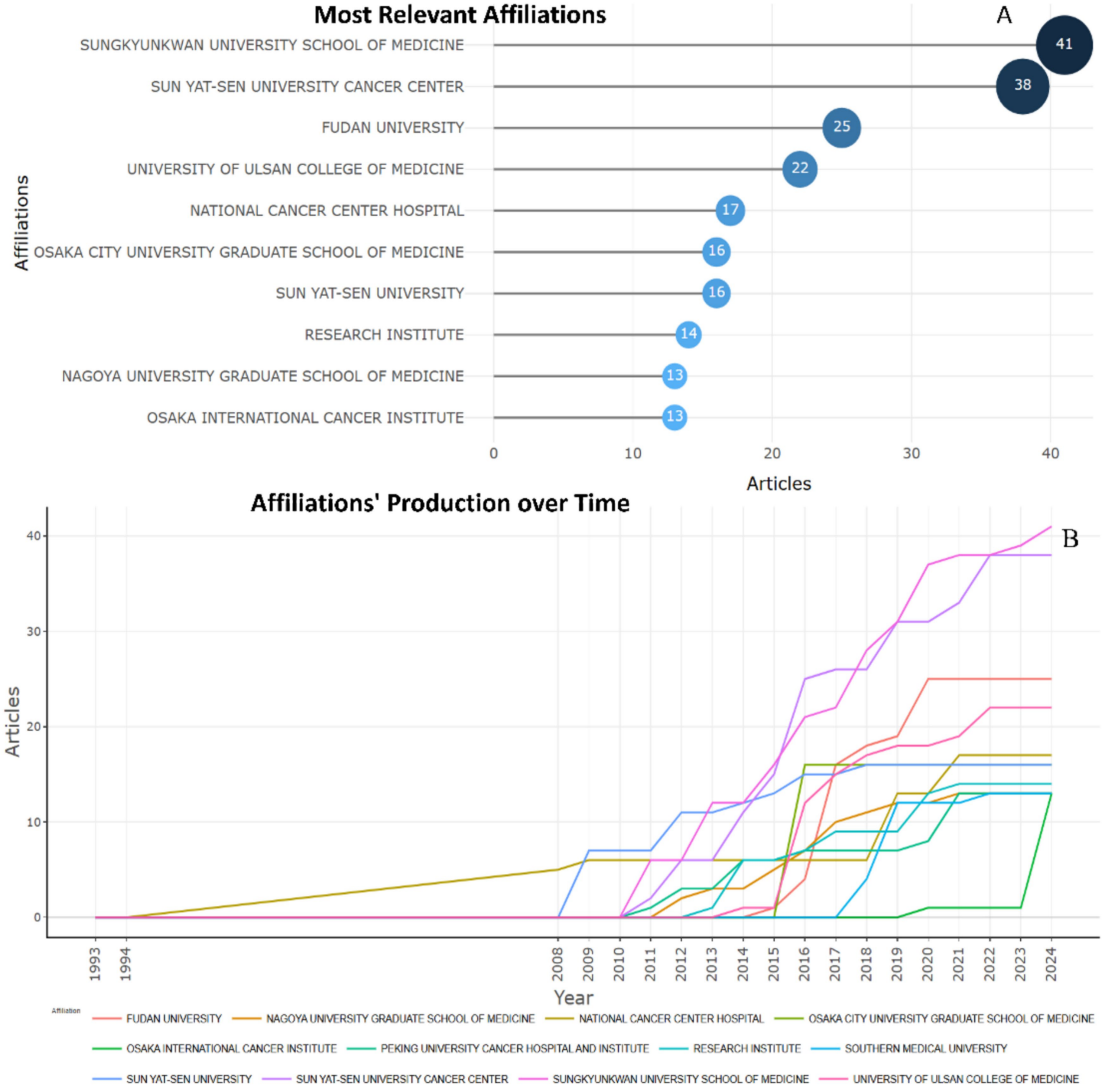
Most relevant affiliations of published research articles of biomarkers used in diagnosis and prognosis of gastric cancer in young patients **(A)** Top 10 largest organizations. **(B)** Top 10 affiliations’ production over time.

In the study of biomarkers used in the diagnosis and prognosis of gastric cancer in young patients, the most productive institutions are SONGKYUNKWAN UNIVERSITY SCHOOL OF MEDICINE and SUNYAT-SEN UNIVERSITY CANCER CENTER, which published 41 and 38 articles, respectively, on this topic (Figure 3A). These institutions have maintained interest in studying biomarkers used in the diagnosis and prognosis of gastric cancer in young patients for many years (Figure 3B).

**Figure 4:**
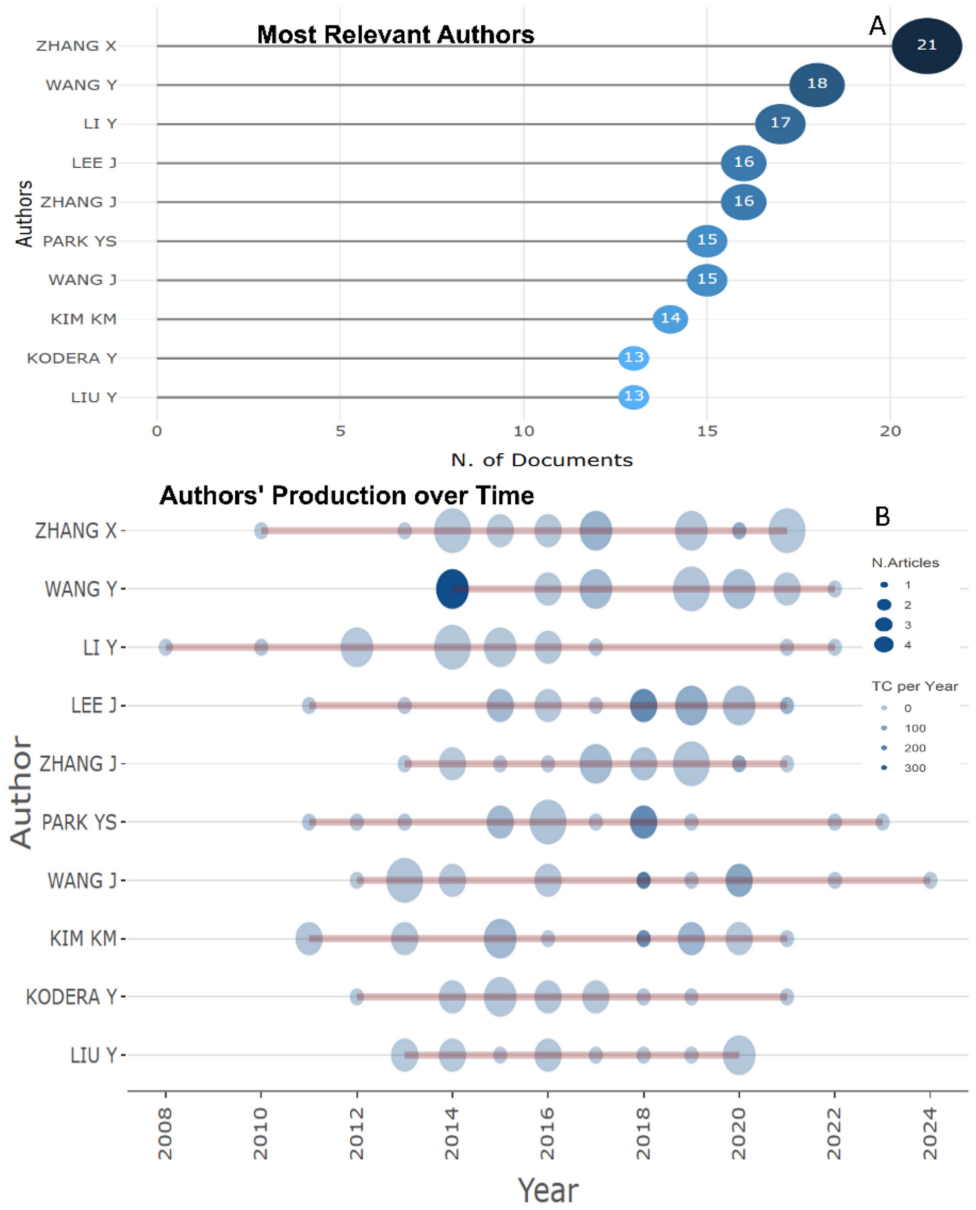
Most productive researchers who published research articles of biomarkers used in the diagnosis and prognosis of gastric cancer in young patients recently. **(A)** Top 10 authors. **(B)** Top 10 prolific authors and publication activity over time (2008–2024).

Among the most productive researchers in the field of studying the biomarkers used in the diagnosis and prognosis of gastric cancer in young patients, three leaders can be distinguished in studies: Zhang X. (21 publications), Wang Y. (18 publications), Li Y. (17 publications) (Figure 4A). Two authors: Zhang X., Wang Y. demonstrate publishing productivity over time (Figure 4B).

**Figure 5:**
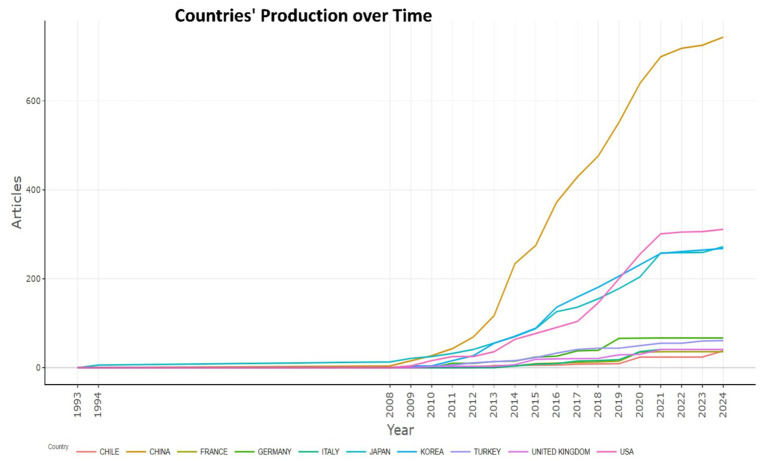
Countries’ production over time of biomarkers used in diagnosis and prognosis of gastric cancer in young patients.

China leads in scientific productivity with 743 publications, illustrating the dominance and academic influence in the field of research of biomarkers used in the diagnosis and prognosis of gastric cancer in young patients. The second is the United States with 311 publications, and the third most prolific is Japan with 272 publications (Table 4). China’s productivity has also grown exponentially over the years, as illustrated in Figure 5.

**Table 4:** Top 10 countries’ scientific production publishing articles of biomarkers used in the diagnosis and prognosis of gastric cancer in young patients.

Ranks	Country	Number of documents
1	China	743
2	USA	311
3	Japan	272
4	South Korea	268
5	Germany	67
6	Turkey	61
7	UK	41
8	Italy	41
9	Chile	38
10	France	36

**Figure 6:**
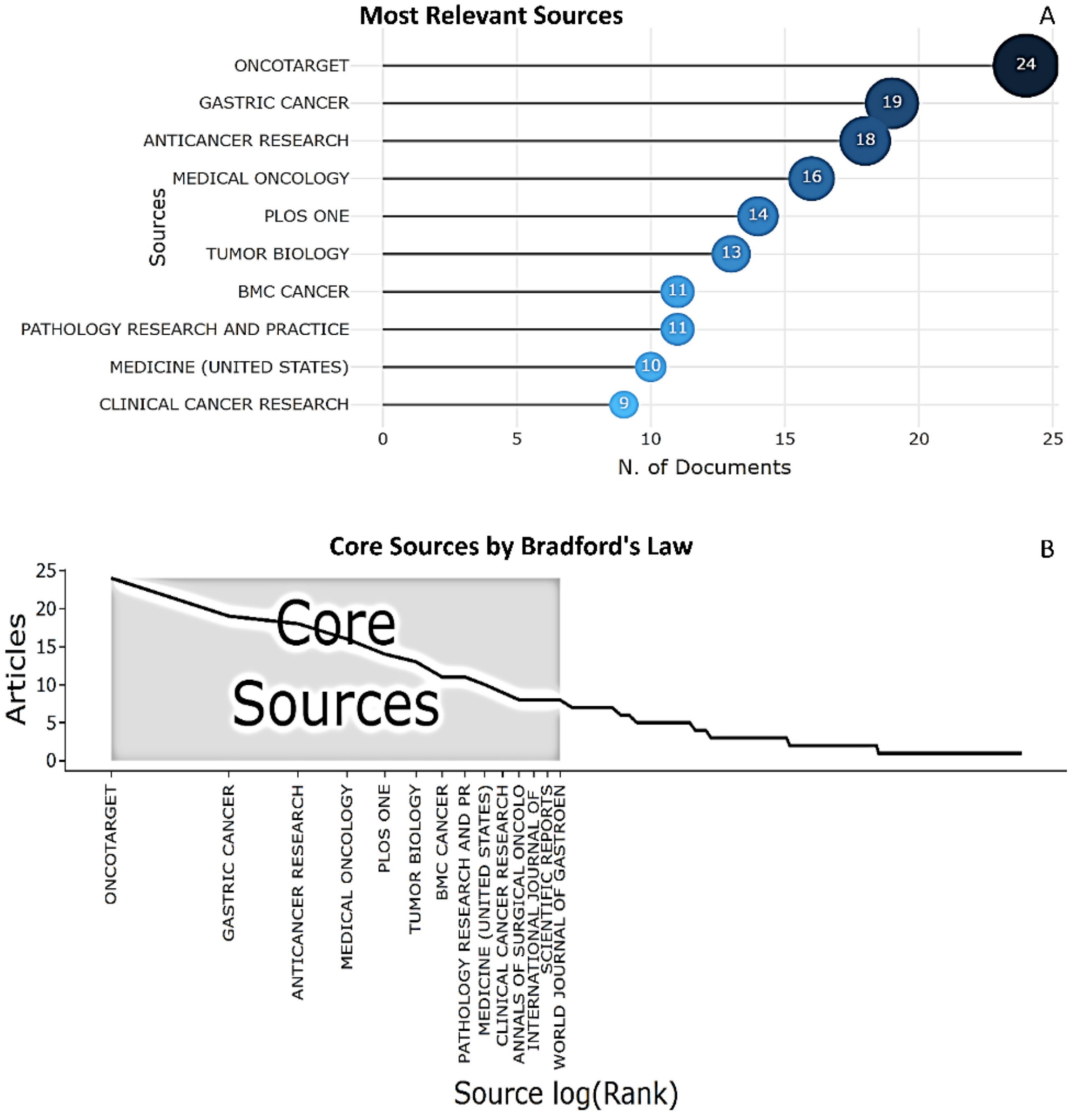
The top 10 most actively publishing journals that published articles regarding biomarkers used in the diagnosis and prognosis of gastric cancer in young patients (1993–2024). **(A)** Top 10 productive journals. **(B)** Broadford’s law plot.

We identified the top 10 most actively publishing journals, among which the top three are: Oncotarget (24 publications), Gastric cancer (19 publications), Anticancer research (18 publications) (Figure 6A). Using the Bradford’s law, we identified 14 main sources that contain the largest number of publications of biomarkers used in the diagnosis and prognosis of gastric cancer in young patients from 1993 to 2024 (Figure 6B).

### 3.4 Global research collaboration

**Figure 7:**
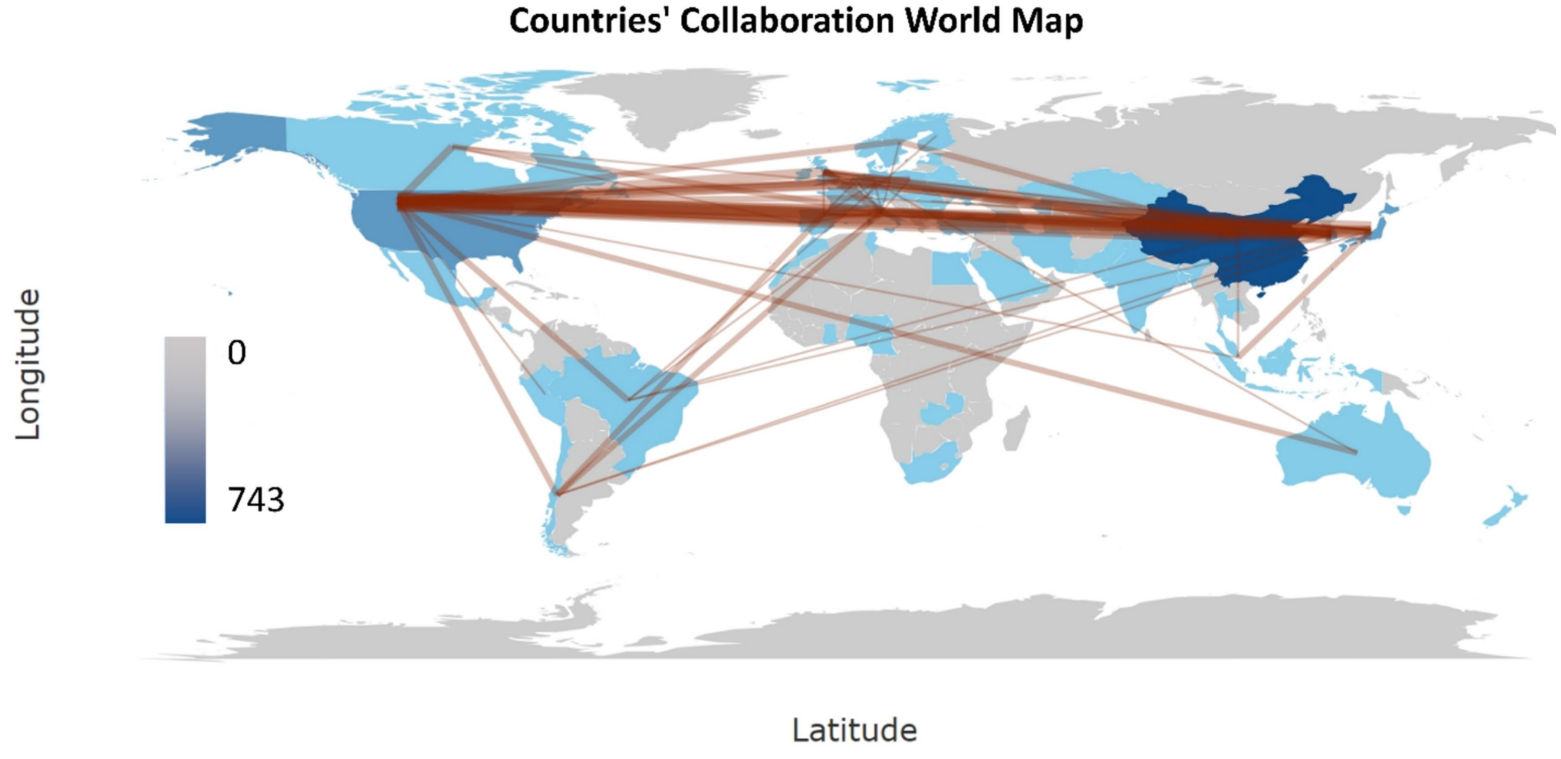
Map of global collaboration of biomarkers used in diagnosis and prognosis of gastric cancer in young patients (1993–2024). The intensity of color saturation corresponds to the increasing number of articles in each country. Collaboration between countries is shown by the thickness of the connecting arrows.

The global collaboration map shows the most productive collaborations between the US and China (22 publications), the US and Korea (18 publications), and the US and Japan (17 publications), illustrating the dominant position of the US as the country of choice for collaboration in research of biomarkers used in the diagnosis and prognosis of gastric cancer in young patients (Figure 7).

**Figure 8:**
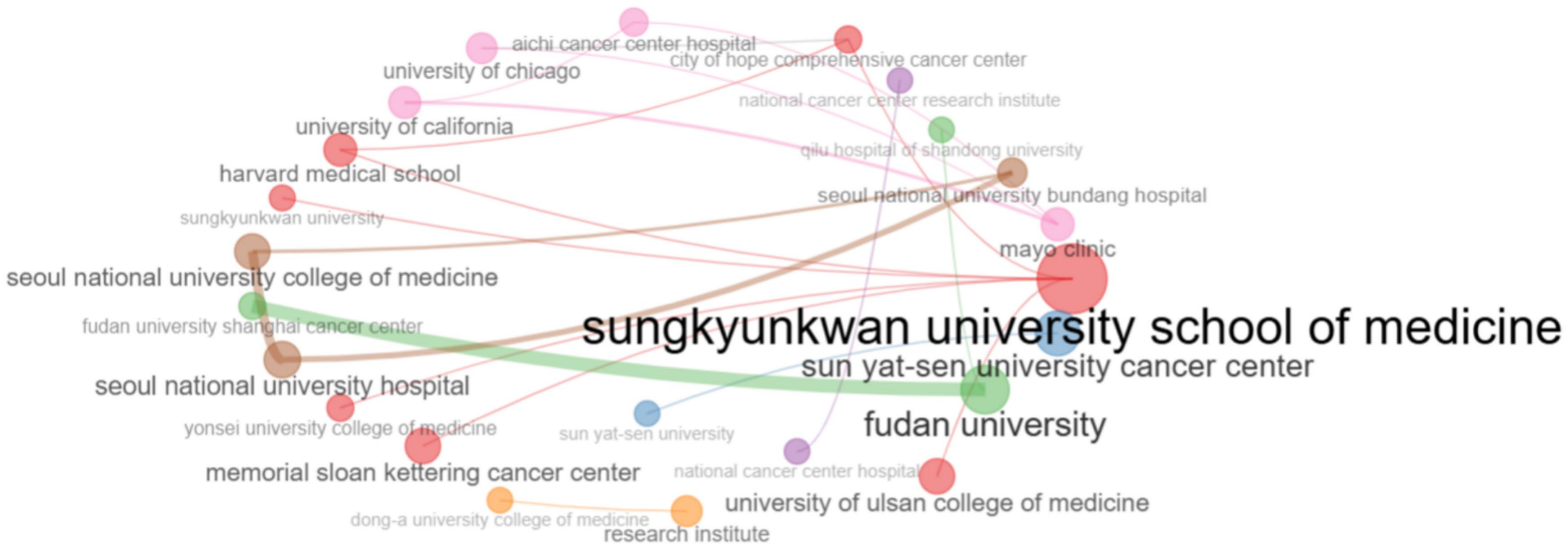
The institutional collaboration network identifies research centers or centers of excellence in research of biomarkers used in the diagnosis and prognosis of gastric cancer in young patients.

A collaboration network has been established to identify central institutions, peripheral institutions and key clusters. As the collaboration network shows Sungkyunkwan University School of Medicine cooperates with a large number of both local and foreign organizations (Figure 8).

**Figure 9:**
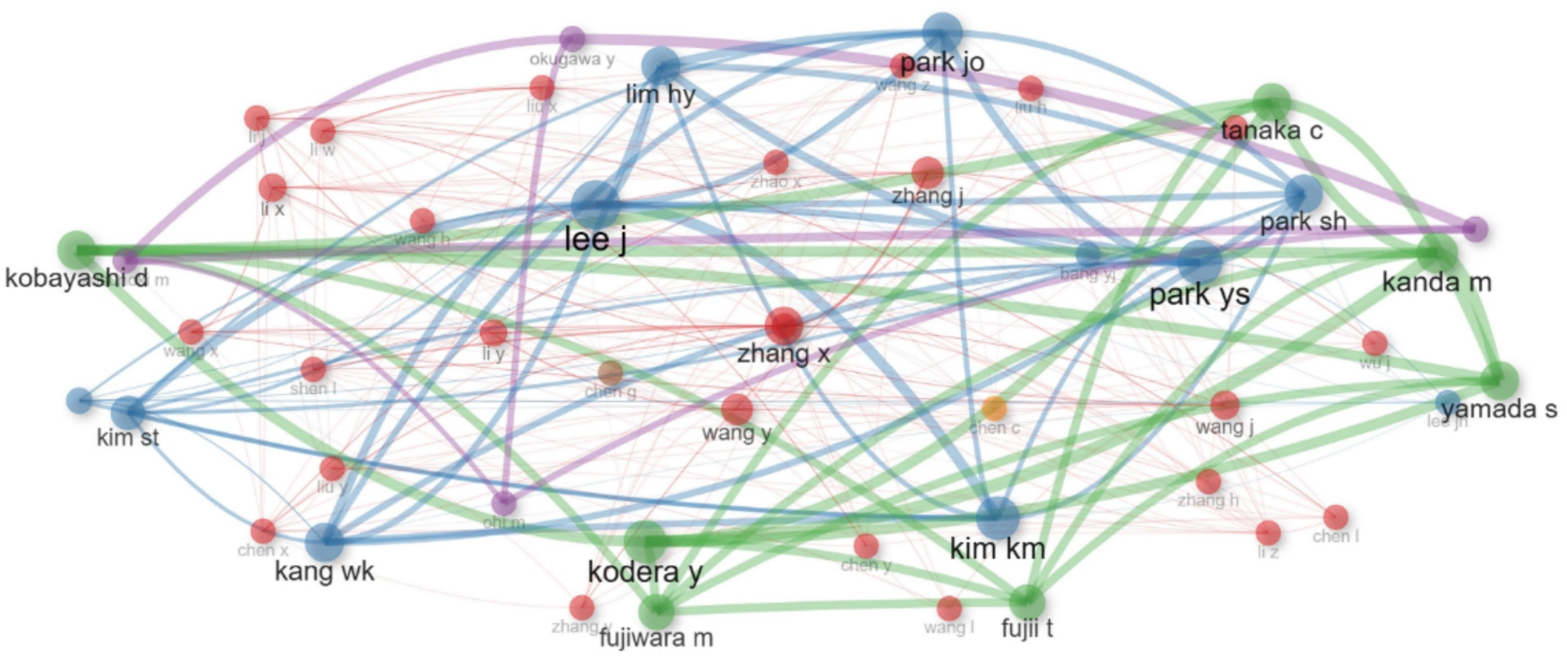
Authors’ collaboration network in research of biomarkers used in the diagnosis and prognosis of gastric cancer in young patients.

The author collaboration network illustrates the key author players, their influence and centrality. Li X. and Zhang X. are leaders who play key roles in the academic environment through whom important communications pass. Authors with lower metric values, such as Wang X., Liu Y. and Wang Z., have more specialized or less influential positions (Figure 9).

### 3.5 Trending keywords

**Figure 10:**
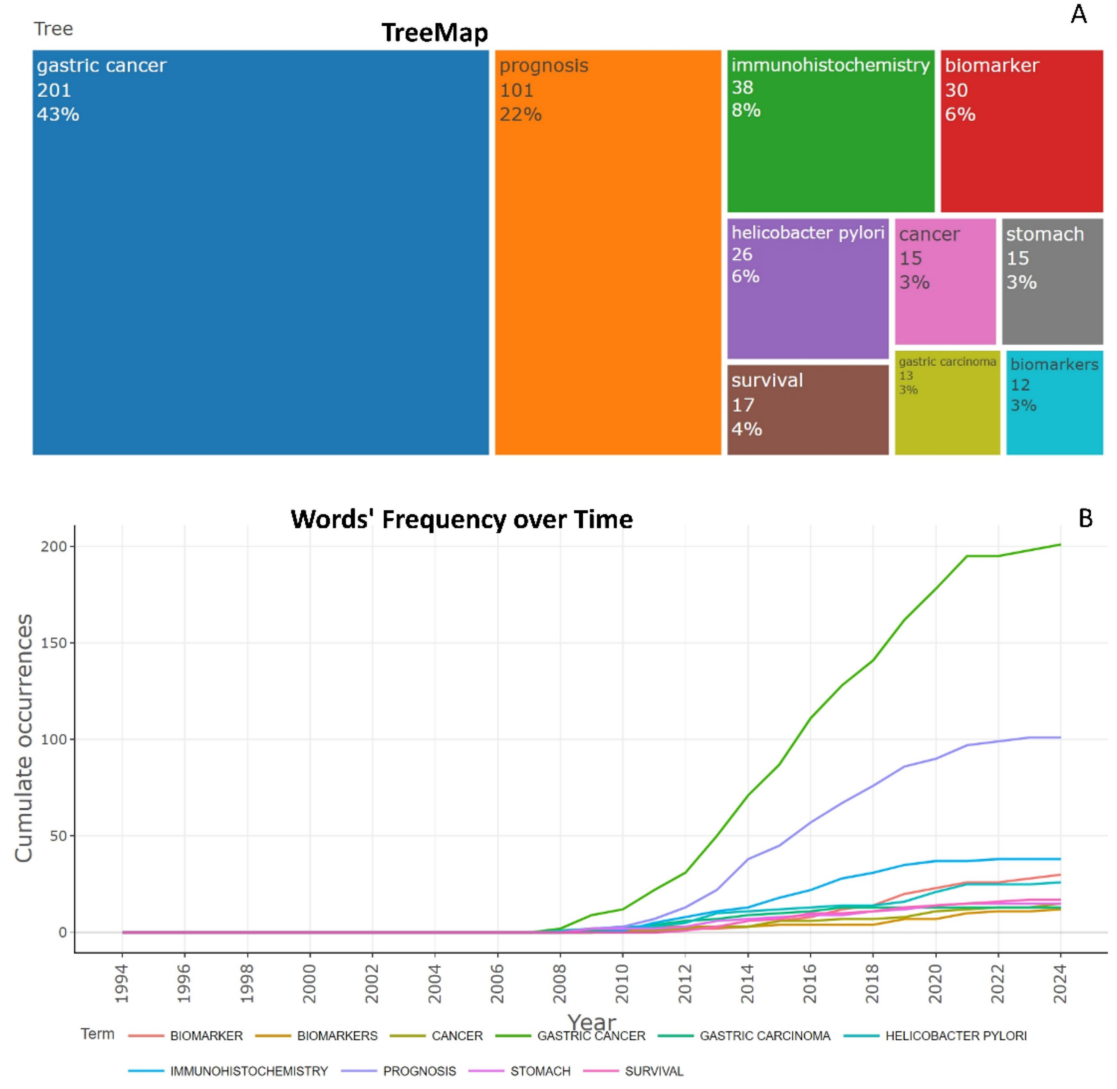
The 10 most popular author’s keywords in studies of biomarkers used in the diagnosis and prognosis of gastric cancer in young patients from 1993 to 2024. **(A)** Tree map and **(B)** scatterplot-Words’ Frequency over Time.

To identify research trends, main topics and directions in scientific literature, keyword analysis was conducted. The thematic map of the 10 most popular keywords reveals significant insights into research focus and trends in the field of biomarkers used in the diagnosis and prognosis of gastric cancer in young patients (Figure 10A). This map provides valuable insights into the research focus and trends in gastric cancer. Dominant topic “Gastric cancer” (201/43%) is the main focus, highlighting the key link position. The second placed topic “Prognosis” (101/22%) indicates a strong focus on research conducted to better understand the outcomes, complications and progression of gastric cancer, finding prognostic biomarkers, which are crucial for diagnosis, patient management and therapeutic approaches. The third most rated topic “Immunohistochemistry” (38/8%) indicates the widespread use of this diagnostic method to detect specific markers. The relatively frequent mention of the topics “biomarker” and “biomarkers” under different spellings (30/6% and 12/3%, respectively) highlights the importance of molecular and genetic markers in the diagnosis of gastric cancer in young patients, prognosis and targeted therapy.

Analysis of word frequency over time in the dataset reflects the evolving research interest in various keywords related to biomarkers used to diagnose and predict prognosis of gastric cancer in young patients (Figure 10B).

The keyword “Gastric cancer” was absent until 2008, marking the beginning of systematic research interest. Since 2008, its usage has steadily increased, with an exponential growth from 50 mentions in 2013 to 201 by 2024, highlighting the growing global recognition of gastric cancer as an important public health problem. “Prognosis,” which first appeared in 2009, has shown a steady upward trend, reaching 101 mentions by 2024. This reflects the sustained interest in predicting outcomes, complications, survival, and improving treatment strategies in gastric cancer. “Immunohistochemistry” with one mention in 2010, the frequency increased to 38 mentions by 2024. This trend highlights the increasing reliance on advanced diagnostic methods in gastric cancer research. “Biomarker” and “Biomarkers” these keywords were rarely mentioned before 2012 but experienced a noticeable surge after 2015. This trend suggests an increasing focus on molecular markers for diagnosis, therapy and prognosis. The combined frequency of “Biomarker” + “Biomarkers” reaches 42 mentions in 2024, highlighting the importance of this research method. This progression reflects the dynamic nature of research priorities and the expanding scope of biomarker research in the context of gastric cancer.

**Figure 11:**
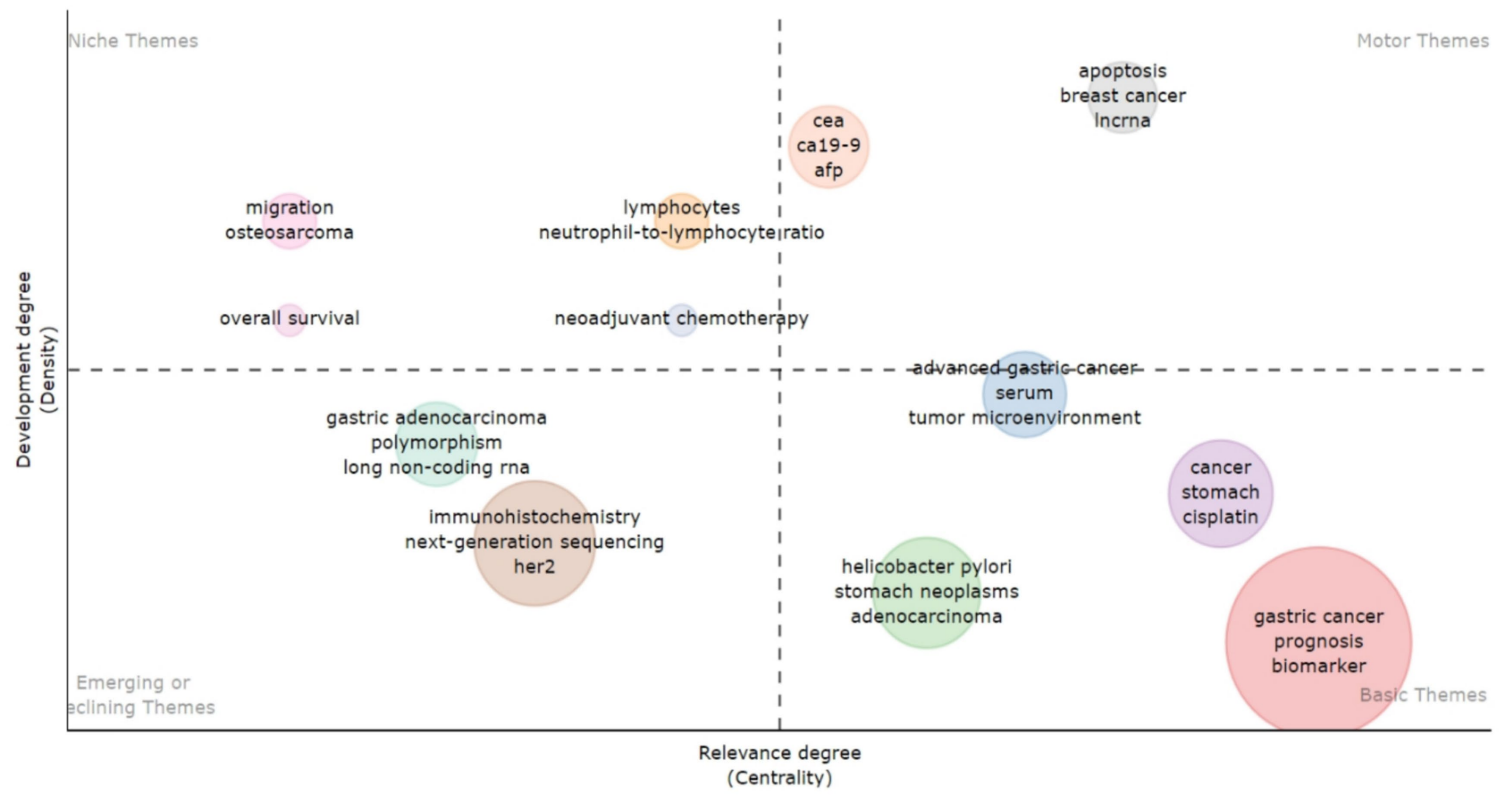
Visualization of thematic map based on author’s keywords frequently used in research articles demonstrates recurring terms and their thematic significance in the context of studying biomarkers used in the diagnosis and prognosis of gastric cancer in young patients.

The analysis of the Topical Map of studies on biomarkers used in the diagnosis and prognosis of gastric cancer in young patients shows that the central theme is “Gastric cancer.” This term serves as the main focus, combining key aspects such as prognosis, biomarkers, treatments (e.g., chemotherapy) and biological processes including DNA methylation. The high frequency of mentions confirms the relevance of this topic. The leading diagnostic methods are “immunohistochemistry” and “next generation sequencing.” Their importance is emphasized by the overlap with biomarker-related topics such as HER2. The high centrality and density of the *Helicobacter pylori*-related cluster confirm its key role in the pathogenesis of gastric cancer, especially in the context of gastric neoplasms and adenocarcinoma. Processes such as apoptosis, long non-coding RNAs (lncRNA), and tumor microenvironment show potential for identifying new therapeutic targets. The topics “neoadjuvant chemotherapy” and “overall survival” are less common, indicating the need for further analysis. Their low frequency may be due to gaps in existing data or the need for new studies. Thus, this map provides a holistic view of current research directions and potential growth points for further study of gastric cancer in young patients (Figure 11).

This topic map shows the complex relationships between author keyword-based clusters in the field of biomarker research for diagnosis and prognosis of gastric cancer in young patients.

**Figure 12:**
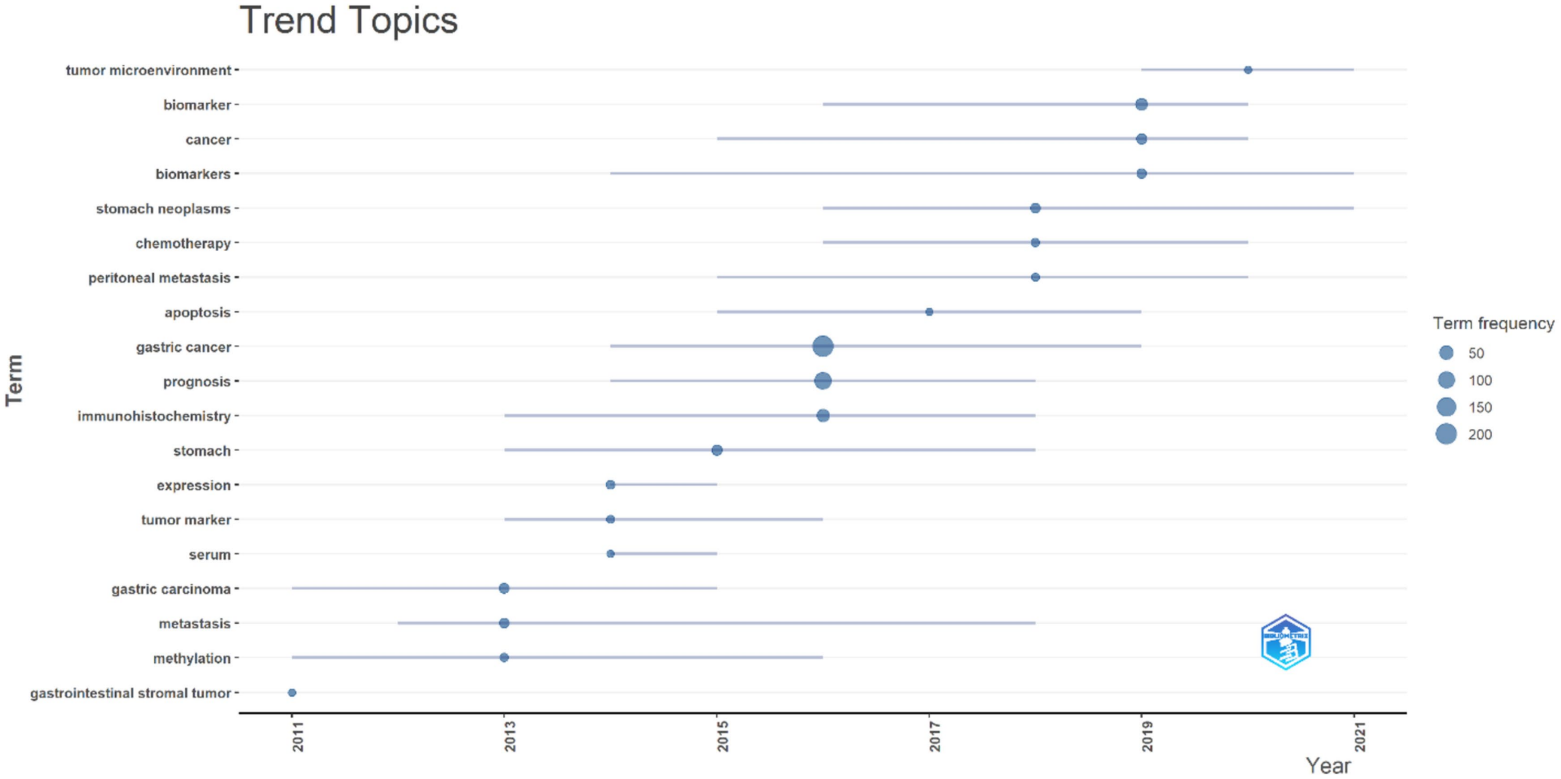
A visualization of the evolution of key topics over time. Each bubble represents the peak frequency of a particular topic, indicating its highest level of relevance or interest in the research community. Connecting lines trace the years during which the topic was actively discussed, illustrating its persistence and overall influence in the field.

The analysis of trending topics shows the temporal evolution and focus of research in the study of biomarkers used for the diagnosis and prognosis of gastric cancer in young patients. The findings provide valuable insight into the emergence, peak activity, and stability of key themes concerning biomarkers used in the diagnosis and prognosis of gastric cancer in young patients (Figure 12).

Terms such as “Prognosis” and “Immunohistochemistry” reached their peak in 2016, reflecting their alignment with diagnostic and prognostic advancements driven by the development of precision medicine during this period. The terms “Expression” and “Serum” peaked in 2014 and exhibited relatively short active periods, emphasizing specific but transient areas of focus in biomarker identification and analysis.

The rising prominence of “Apoptosis,” which peaked in 2017, and “Tumor Microenvironment,” which peaked in 2020, underscores a growing emphasis on molecular processes and their implications for therapeutic strategies. This shift signifies a deeper exploration of the mechanistic foundations of gastric cancer. The terms “Chemotherapy” and “Peritoneal Metastasis,” with median activity around 2018, highlight critical clinical challenges, particularly in managing advanced stages of gastric cancer. “Biomarker” and “Biomarkers,” both with median activity in 2019, underscore the pivotal role of molecular markers in diagnosis, treatment, and prognosis. Their prominence aligns with the increasing adoption of advanced technologies such as next-generation sequencing, which have driven significant progress in the field. Persistent and expanding themes from 2019 to 2021 include “Gastric Neoplasms,” which, with a median activity in 2018, demonstrates both foundational and clinical research dimensions due to its high frequency of usage and extended timeline. Additionally, “Tumor Microenvironment,” although lower in frequency, reflects a recent surge in interest as a potential therapeutic target, marking it as an emerging area of importance. This analysis provides a comprehensive view of research trends, highlighting the dynamic evolution of focus areas in gastric cancer biomarker studies.

## 4 Discussion

As the clinical application of biomarkers improves, understanding the biology and treatment of gastric cancer becomes more complex. Biomarker-based treatment approaches provide more personalized care for gastric cancer (20). The introduction of immuno-oncology concepts has opened a new era in the diagnosis, treatment and prognosis of gastric cancer (21).

Modern advances in gastric cancer therapy are accompanied by active identification of new biomarkers associated with various cellular pathways and advanced diagnostic technologies. Standardization of clinical protocols and technological improvement of biomarker assessment methods are expected to facilitate a more personalized approach to treatment and further enhance the effectiveness of therapeutic outcomes (21). As of 2021, it was established that three molecular biomarkers predict response to targeted therapies in gastric and gastroesophageal junction (G/GEJ) cancer: HER2 positivity for trastuzumab and trastuzumab deruxtecan, and PD-L1 expression for pembrolizumab and microsatellite instability (MSI) status (22). Chen X. et al. found that eXosomal PD-L1 (exoPD-L1) may contribute to functional immunosuppression, which negatively affects the survival of patients with gastric cancer (23). Shimura T. et al. showed that m6A regulators may become promising prognostic biomarkers in gastric cancer (24).

Identifying key changes and biomarkers that can predict the efficacy of immunotherapy based on a limited number of samples is a critical task (25). Zhuo Hanet et al. in their study studied the relationship between the level of PD-L1 expression and clinical characteristics. They found that age (>60 years), tumor size (>5 cm), EBV infection (+), Her-2 expression (+), microsatellite status (MSI), and mismatch repair status (dMMR) were risk factors for positive expression of PD-L1 in gastric cancer (26). The study of the relationship between PD-L1 and HER2 and the clinicopathological features of gastric cancer and its prognostic effect revealed that TNM stage and PD-L1 expression were independent prognostic factors for survival time. PD-L1 expression has biological significance in gastric cancer and is closely related to the clinicopathological characteristics and prognosis (27). The study of prognostic biomarkers of gastric cancer for a subgroup of young patients (>40) is an important direction for identification of key markers that may facilitate the development of new therapeutic approaches. This will make it possible to reduce premature morbidity and mortality associated with gastric cancer at a young age. Today there is a lack of data regarding the study of biomarkers, clinical characteristics and prognostic factors for stomach cancer in young people (6). This was also confirmed by the paucity of studies examining biomarkers used to diagnose and predict gastric cancer specifically in young patients.

This bibliometric analysis provides a comprehensive overview of studies on biomarkers used for diagnosis and prognosis of gastric cancer (GC) in young patients. Results show significant fluctuations in both publication and citation trends over a 31-year period, highlighting evolving research interests and results. A consistent 7.71% annual growth indicates sustained scientific interest in biomarkers for gastric cancer in young patients. The low rate of international co-authorship (19.4%), highlighting the need for greater global partnerships. Articles by US authors have a high average citation rate (53.56 citations per year). Publications were particularly concentrated in 2014 (59articles) and 2020 (57articles), likely due to advances in molecular biology and genomics that have stimulated interest in GC biomarkers. A steady decline in citations since 2015, suggest a saturation in foundational research and fewer high-impact publications in recent years. The 2014 article on anti-PD-L1 antibody by Herbst et al. was the most cited, emphasizing the importance of therapeutic innovations like immune agents. The study identifies Sungkyunkwan University School of Medicine, Sun Yat-sen University School of Cancer Center and Fudan University, as the most prolific institutions, highlighting the concentration of research efforts in East Asia. These institutions have been key contributors, generating much of the literature regarding biomarkers used in the diagnosis and prognosis of gastric cancer in young patients. Central figures like Li X. and Zhang X. facilitate influential collaborations. The top three countries in terms of productivity were (1) China with 743 publications; (2) USA with 311 publications; (3) Japan with 272 publications. Their leadership in stomach cancer research reflects a combination of epidemiological burden, investment in science, and global collaboration. The analysis also emphasizes the importance of international collaboration in advancing this research, especially between institutions in East Asia, North America, and Europe.

However, a critical gap in the number of studies specifically targeting young patients with GC is evident, highlighting the need for more targeted research. Despite the progress made in molecular biomarker research, citation rates have dropped sharply from 2018 to 2024, suggesting a slowdown in this field. This decline may reflect difficulties in translating molecular discoveries into clinical applications or changing research priorities.

Co-citation analysis classified studies into distinct intellectual groups, identifying key themes and areas of focus in GC biomarker research. This structure may help identify emerging topics and guide future research. In addition, significant differences in citation rates were noted across regions, with studies from the US receiving the highest citation rates, indicating regional differences in research impact. The strongest research partnerships exist between the USA and China, followed by the USA with Korea and Japan. Central figures like Li X. and Zhang X. facilitate influential collaborations.

Analysis of key terms such as “gastric cancer,” “prognosis” and “biomarkers” indicates the dominant importance of clinical issues related to disease outcomes and the development of precision medicine. After 2020, there is a stabilization or slowdown in the frequency of mention of these terms, which may indicate maturity in certain research areas. Significant growth in interest in keywords related to diagnostics (immunohistochemistry, biomarkers) and outcomes (prognosis, survival) reflects a shift in research focus toward personalized medicine. Simultaneously, the moderate growth of terms such as “gastric carcinoma” and “gastric” indicates their secondary importance in the context of specialized research.

The present analysis demonstrates the dynamic development and major research directions of biomarkers used for the diagnosis and prognosis of gastric cancer, especially in young patients. It also suggests strategic approaches for further research, including a focus on prognostic markers, the role of the tumor microenvironment, and *Helicobacter pylori*-associated pathogenesis.

The thematic review emphasizes the promise of integrating diagnostic technologies and data on apoptosis processes with molecular studies to develop new treatments and improve patient survival rates. The temporal dynamics of the studies indicate a shift from more general topics such as “gastric carcinoma” and “gastric” to specialized aspects including the study of biomarkers, tumor microenvironment and methylation processes. Early work focused on identifying the underlying pathologic mechanisms, whereas current trends emphasize molecular mechanisms and therapeutic innovations.

### 4.1 Strengths and limitations

This study offers several notable advantages. It represents the first comprehensive bibliometric analysis systematically examining the global landscape of research of biomarkers used in the diagnosis and prognosis of gastric cancer in young patients over the past 31 years. We performed a thorough and consistent analysis, presenting and interpreting metadata using a widely accepted and reliable software platform. However, there are some limitations that should be considered. Our analysis is based solely on data from the Scopus database and focuses exclusively on research articles, which may lead to a potential underestimation of publications indexed in other databases. The focus on research articles means that reviews and other forms of research papers were not included in the analysis.

In addition, the database used includes a limited number of studies related to the keywords “gastric cancer,” “biomarkers,” and “young adults.” This limitation may lead to an incomplete representation of the research landscape in this field, highlighting the need for further exploration of other data sources and research areas.

Future studies should examine a wider range of databases and include a wider range of publications to obtain a more holistic view of the research landscape.

## 5 Conclusion

The growth of publications, high citation rates and participation of researchers from many countries confirm the importance of research devoted to the biomarkers used in the diagnosis and prognosis of gastric cancer in young patients. However, despite significant progress, this bibliometric analysis demonstrates the need for more targeted research in younger patients. Identifying age-specific biomarkers will be critical to improving diagnosis, prognosis, and personalized treatment strategies for young patients with gastric cancer. The study highlights the need for global collaboration and multidisciplinary approaches to drive the next wave of breakthroughs in this field. Tumor microenvironment, molecular diagnostics, immune agents, and advanced sequencing methods present promising future directions. Greater focus on underexplored topics like neoadjuvant chemotherapy and long-term survival outcomes is needed to fill existing gaps.

## Data Availability

The original contributions presented in the study are included in the article/supplementary material, further inquiries can be directed to the corresponding author.
